# ANTXR1 Regulates Erythroid Cell Proliferation and Differentiation through wnt/*β*-Catenin Signaling Pathway *In Vitro* and in Hematopoietic Stem Cell

**DOI:** 10.1155/2022/1226697

**Published:** 2022-08-27

**Authors:** Tingting Jin, Zhaojun Zhang, Yuanyuan Han, Di Li, Juan Liu, Minmin Jiang, Ryo Kurita, Yukio Nakamura, Fangfang Hu, Xiangdong Fang, Shengwen Huang, Zhaolin Sun

**Affiliations:** ^1^School of Medicine, Guizhou University, Guiyang, Guizhou 550025, China; ^2^Prenatal Diagnosis Center, Guizhou Provincial People's Hospital, Guiyang, Guizhou 550002, China; ^3^CAS Key Laboratory of Genome Sciences and Information, Beijing Institute of Genomics, Chinese Academy of Sciences, Beijing 100101, China; ^4^Cell Engineering Division, RIKEN Bio Resource Center, Tsukuba, Ibaraki 305-0074, Japan; ^5^Department of Laboratory, Guizhou Provincial People's Hospital, Guiyang, Guizhou 550002, China; ^6^NHC Key Laboratory of Pulmonary Immunological Diseases, Guizhou Provincial People's Hospital, Guiyang, Guizhou 550002, China; ^7^Department of Urology, Guizhou Provincial People's Hospital, Guiyang, Guizhou 550002, China

## Abstract

Erythropoiesis is a highly complex and sophisticated multistage process regulated by many transcription factors, as well as noncoding RNAs. Anthrax toxin receptor 1 (ANTXR1) is a type I transmembrane protein that binds the anthrax toxin ligands and mediates the entry of its toxic part into cells. It also functions as a receptor for the Protective antigen (PA) of anthrax toxin, and mediates the entry of Edema factor (EF) and Lethal factor (LF) into the cytoplasm of target cells and exerts their toxicity. Previous research has shown that ANTXR1 inhibits the expression of *γ*-globin during the differentiation of erythroid cells. However, the effect on erythropoiesis from a cellular perspective has not been fully determined. This study examined the role of ANTXR1 on erythropoiesis using K562 and HUDEP-2 cell lines as well as cord blood CD34^+^ cells. Our study has shown that overexpression of ANTXR1 can positively regulate erythrocyte proliferation, as well as inhibit GATA1 and ALAS2 expression, differentiation, and apoptosis in K562 cells and hematopoietic stem cells. ANTXR1 knockdown inhibited proliferation, promoted GATA1 and ALAS2 expression, accelerated erythrocyte differentiation and apoptosis, and promoted erythrocyte maturation. Our study also showed that ANTXR1 may regulate the proliferation and differentiation of hematopoietic cells, though the Wnt/*β*-catenin pathway, which may help to establish a possible therapeutic target for the treatment of blood disorders.

## 1. Introduction

Hematopoietic stem cells (HSCs) can self-renew and differentiate into erythroid lines [1]. By dividing into megakaryocytic erythroid progenitors (MEPs) through common myeloid progenitor cells (CMPs) and erythroblasts through erythroid blast-forming units (BFU-E) and erythroid colony-forming units (CFU-E), HSCs form reticulocytes through the terminal differentiation stage, and then enter the bloodstream and become mature red blood cells, highly specialized functional cells. In this process, new erythrocytes are continuously generated, and the spontaneous elimination of senescent erythrocytes maintains a dynamic balance. Blood disorders such as sickle cell anemia, *β*-thalassemia, and sideroblastic anemia are often caused by red blood cells with abnormal morphology and function [2–4]. Life-long blood transfusions or bone marrow transplantation may be required in severe cases of blood diseases with complications. Therefore, promoting the production of effective red blood cells is an effective treatment for hemolytic anemia. Previous studies have shown that erythropoiesis is regulated by many microenvironmental factors, such as miRNAs and transcription factors, particularly the crucial GATA1.

Initially, Krüppel-like factor 1 (KLF1 or EKLF, erythroid Krüppel-like factor) and GATA1 primitively and definitively regulate erythropoiesis under physiological and pathological conditions by targeting different erythroid-specific genes [5], but their regulatory networks remain unclear. Anthrax toxin receptor 1 (ANTXR1) is a membrane protein discovered 20 years ago, and it plays an important role in extracellular matrix homeostasis, angiogenesis, and cell proliferation [6, 7]. A recent study has found that ANTXR1 is a target of Runx2 and regulates the proliferation and apoptosis of chondrocytes [8]. The role of ANTXR1 in tumor development has been demonstrated in previous studies, but no studies have examined its effects on hematopoiesis and erythrocyte differentiation. Until now, two single-nucleotide polymorphisms (SNPs), known as rs4527238 and rs35685045, have been associated with the expression of fetal hemoglobin (HbF) in patients with sickle cell anemia [9–11]. We overexpressed and interfered with ANTXR1 in K562 cells, cord blood CD34^+^ cells, and HUDEP-2 cells in the early stage and found that the expression of the *γ*-globin gene was inversely proportional to the expression of the ANTXR1 gene, and interfering with the gene inhibited cell proliferation.

Canonical Wnt signaling has been implicated in the regulation of hematopoiesis. Wnt signaling pathways play important roles in self-renewal of hematopoietic stem cells [12]. The knockout of *β*-catenin in the mouse hematopoietic system results in impaired HSCs self-renewal [13]. HSCs self-renewal is impaired, and hematopoietic reconstitution capacity is reduced when Wnt inhibitors, such as Axin1, DKK1, and Wif1, are overexpressed in bone marrow stromal cells and osteoblasts [14–16]. There is evidence that hematopoietic stem cells and their hematopoietic microenvironment can receive and respond to Wnt signaling. However, the role of ANTXR1 in regulating erythroid cell proliferation and differentiation through the Wnt/*β*-catenin signaling pathway has not been proven. In this study, differentiated erythroid cell models were used to investigate the role of ANTXR1 in the proliferation and differentiation of erythroid cells and its possible mechanisms. Based on the findings of this study, we might be able to promote the production of red blood cells that are more effective in the treatment of blood disorders.

## 2. Materials and Methods

### 2.1. Cell culture

K562 cell line was purchased from Shanghai Institute of Biological Sciences, China. Cord blood (CB) samples were obtained from the Obstetrics Department of Beijing Maternity Hospital. The institutional ethical committee of Guizhou Provincial People's Hospital approved the study. All methods were conducted following Declaration of Helsinki guidelines and regulations. HUDEP-2 cells were donated by RIKEN Tsukuba Branch, Ibaraki, Japan.

K562 cells were cultured in RPMI-1640 medium containing 10% fetal bovine serum (FBS) and 1% streptomycin/penicillin. The culture media was changed once daily and passaged once every two days. The erythroid differentiation of K562 was induced for three days with 50 *μ*mol/L hemin and cultured in a 37°C, 5% CO2 incubator.

Isolated CD34^+^ cells (STEMCELL,#17896) were first plated in StemSpan SFEM II (STEMCELL Technologies) supplemented with 50 ng/mL SCF, 50 ng/mL, Flt3 ligand, 50 ng/mL TPO, and 2% penicillin-streptomycin for 7 days according to the manufacturer's instructions. On day 7, the cells were cultured to promote erythroid differentiation using conditions modified from those reported previously [10]. The cells were inoculated for 7 days at a density of 5 × 10^5^/mL in StemSpan SFEM II medium containing 10 ng/mL SCF, 10 ng/mL IL-3, 3 IU/mL EPO, and 2% penicillin-streptomycin, followed by the addition of 10 ng/mL SCF, 3 IU/mL EPO, and 2% penicillin-streptomycin and culture for a further 3 days. 1 IU/mL EPO and 2% penicillin-streptomycin were then added continually to the SFEM II medium for a further 6 days, resulting in a total incubation time of 16 days.

Culture of the HUDEP-2 cells for erythroid differentiation involved: (1) transfer of the cells to the erythroid differentiation medium (EDM) containing Iscove's modified Dulbecco's medium (IMDM), 2% penicillin-streptomycin solution (10,000 U/mL stock concentration), 500 *μ*g/mL human holo-transferrin, 10 *μ*g/mL recombinant human insulin solution, 3 IU/mL heparin, 3% inactivated human plasma, and 3 IU/mL Epoetin alfa (Epogen, Amgen); (2) culture for 4 days in EDM containing 100 ng/mL SCF and 1 *μ*g/mL doxycycline; and (3) culture for 3 days in EDM containing 1 *μ*g/mL doxycycline.

### 2.2. Overexpression and Knockdown of ANTXR1-Transfected K562 Cells, Cord Blood CD34^+^, and HUDEP-2 Cells

Semiattached K562 cells were cultured in RPMI 1640 (Gibco) supplemented with 10% FBS (Gibco), penicillin 100 units/mL, and streptomycin 100 *μ*g/mL at 37°C in a humidified 5% CO_2_ incubator overnight. K562 cells were seeded at a density of 5 × 10^5^ cells per well, then transfected with the overexpression (OE) plasmids (titer 1 × 10^8^ transduction unit (TU)/mL, multiplicity of infection MOI = 30) and control plasmids (titer 1 × 10^7^ TU/mL, MOI = 30) transfected into K562 cells, and were cultured with fresh RPMI-1640 medium containing 10% fetal bovine serum for 48 h. The NC-shRNA and ANTXR1-sh5 sequences were designed by online software (https://www.sigmaaldrich.cn/CN/zh/semi-configurators/shrna?activeLink=productSearch). The K562 cells were infected with the shNC (titer 1 × 10^8^ TU/mL, MOI = 20) virus or ANTXR1-sh5 (titer 4 × 10^8^ TU/mL, MOI = 20) virus with Invitrogen Lipofectamine™ 3000 Transfection Reagent following the commercial protocol. On the second day of erythroid differentiation, CD34^+^ cells were infected with the virus, and the culture medium was changed after 8 hours of infection. HUDEP-2 lentivirus infection was the same as K562 cells infection, and GFP-positive cells were screened by flow cytometry.

### 2.3. qRT-PCR

For the detection of mRNA expression, total RNA was reversed and transcribed into cDNA using the 2^-*ΔΔ*Ct^ method. The PCR was carried out at 95°C for 10 min; 95°C for 10s, and 60°C for 30s for 40 cycles. Each sample was repeated 3 times. The gene expression of the *ANTXR1*, *GATA1*, and *ALAS2* was detected by RT-PCR. The primers of the target genes are shown in Table 1.

### 2.4. Flow Cytometry

Approximately 1 × 10^6^ of K562 and CD34^+^ cells were collected and washed twice with 1 × PBS. The cells were then permeabilized using TritonX-100 (Beyotime, #P0096) for 20 minutes. Following immunostaining, antibodies to CD235a-APC (#REA175) and CD71-PE (Miltenyi Company, Germany, #AC102) were added. The cells were incubated for 30 minutes at room temperature in the dark, and fluorescence-activated cell sorting (FACS) machine was then used to analyze CD71 and CD235a expression.

### 2.5. Benzidine Staining

Benzidine hydrochloride powder (10 mg) was dissolved in 1 mL of 0.5 M glacial acetic acid solution. 50 *μ*L of the above solution was added to 1 *μ*L of 30% hydrogen peroxide to prepare a benzidine work solution. About 0.5 million cells were spread on the slide after two washes with 100 *μ*L 1 × PBS and incubated at room temperature for 5 min following addition of 1 *μ*l benzidine solution to the cell suspension. The staining results were observed, and about 200 cells in a field were counted under a microscope. The positive rates of blue-stained cells in 3 different fields were calculated. Benzidine staining was used to determine K562 cell differentiation by synthesizing hemoglobin.

### 2.6. Wright-Giemsa Staining

The entire glass slide area smeared with cells was covered with drip dye solution A (0.8-1.0 mL) and then evenly stained for 1 min. Later, solution B (which has a volume twice that of solution A) was added, washed, and stained for approximately 8 minutes each. The slide was then gently rinsed to remove the residual dye solution, air-dried, and observed under the microscope for the effect of ANTXR1 on K562 cells during erythroid differentiation.

### 2.7. Western Blot

Using a collection of 1 × 10^6^ cells from the overexpression, knockdown, and control groups, western blotting was used to determine protein expression in transfected cells. In brief, the cells were washed three times with PBS, and 200 *μ*L of protein lysis buffer was added to extract the total protein. The protein concentration was determined with the BCA protein assay. After loading 20 *μ*g total protein in the SDS-PAGE, it was run for 5 min at 95°C using protein sample buffer. The protein was transferred into a PVDF membrane through electrophoresis at 120 V for 2 h. The membrane was then blocked with 5% bovine serum albumin for 1 h at room temperature. The primary ANTXR1 antibody of the target gene 1 : 1000 (ab21270, Abcam), anti-phospho-*β*-catenin (Ser33/37/Thr41) 1 : 1000 (ab246504, Abcam), anti-*β*-catenin 1 : 2000 (#8480, CST), anti-GATA1 1 : 1000 (ab133274, Abcam), anti-ALAS2 1 : 1000 (ab184964, Abcam), anti-GAPDH 1 : 3000 (ab181602, Abcam), and anti-GSK3*β*1:3000 (ab32391, Abcam) was added, respectively, for each detection and incubated overnight at 4°C. The membrane was washed thrice with TBST, 5 min each time, and incubated with a secondary antibody for 1 h at room temperature. The membrane was then re-washed thrice with TBST, and the band was developed with ECL Iuminescence solution. Finally, immunoreactive protein bands were visualized using the ECL system, and the protein bands were analyzed using Image J software (http://imagej.nih.gov/ij).

### 2.8. Cell Proliferation Assay

We aimed to detect the effect of ANTXR1 on the proliferation of K562 cells and CD34^+^ cells. Cell proliferation assay was done using Biyuntian Cell Counting Kit-8 (#C0038, Biyuntian Biotech, Jiangsu, China). The experimental and the control cells were plated into a 96-well plate at a density of 1 × 10^4^ cells/well, and the K562 cell line were divided into groups at 12, 24, 48, and 72 hours according to protocol; 10 *μ*L of CCK-8 solution was added to the well plates at various times, and the OD absorbance value of each well at 450 nm was detected by a microplate reader at 12, 24, 48 and 72 hours. Cord blood CD34^+^ cells were evaluated for cell proliferation on D6, D7, D8, and D9.

### 2.9. Apoptosis Assay

The apoptosis experiment was performed with an Annexin V-PE apoptosis detection kit (#C1065S, Biyuntian, Jiangsu, China.). The cord blood CD34^+^ cells of the experimental and the control group were centrifuged at 300*g* to remove the supernatant. The cells were then washed with 1 × PBS, and the supernatant was discarded. Later, 195 *μ*L Annexin V-PE binding solution was added, and the cells were gently resuspended before adding 5 *μ*L Annexin V-PE with gentle mixing. The mixture was incubated in the dark at room temperature for 15 minutes, washed with 1 × PBS, resuspended in 500 *μ*L of 1 × PBS, and placed on ice for flow cytometry detection.

### 2.10. Immunofluorescence Staining

To detect the localization of ANTXR1 and LRP6 in K562 cell line, we collected 1 × 10^6^ cells from the experimental and the control group. The cells were then centrifuged, washed twice with 1 × PBS, and resuspended in 20 *μ*L of 1 × PBS. Cells were later introduced onto glass slides to air dry naturally. The cells were fixed for 30 min using 4% paraformaldehyde. Later, rinsing was done three times with 1 × PBS for 5 min each time before air-drying the cells naturally. Cells were permeabilized with TritonX-100 at room temperature for 15 min and then rinsed thrice with 1 × PBS for 5 min. The samples were then blocked with 5% BSA/PBS solution for 30 min at room temperature before blocking with 50 *μ*L of primary antibodies ANTXR1 1 : 100 (ab21270, Abcam) and LRP6 1 : 100 (ab75358, Abcam). Cells were incubated overnight at 4°C and rinsed thrice with 1 × PBS. Later, 50 *μ*L of fluorescein-labeled secondary antibody (#A-11037, Thermo Fisher Scientific) was added and incubated in the dark for 1 h at room temperature. The samples were then rinsed thrice with 1 × PBS, stained with DAPI, and observed in a laser confocal microscope.

### 2.11. Statistical Analysis

Data were analyzed using SPSS17.0 software and presented as mean ± standard deviation. The experiments were independently repeated three times, and the statistical significance between groups was determined by an independent *t*-test, and ^∗^*P* < 0.05 or ^∗∗^*P* < 0.01 was considered a significant difference.

## 3. Results

### 3.1. ANTXR1 Effects on Erythrocyte Differentiation during Erythroid Differentiation

**Figure 1:**
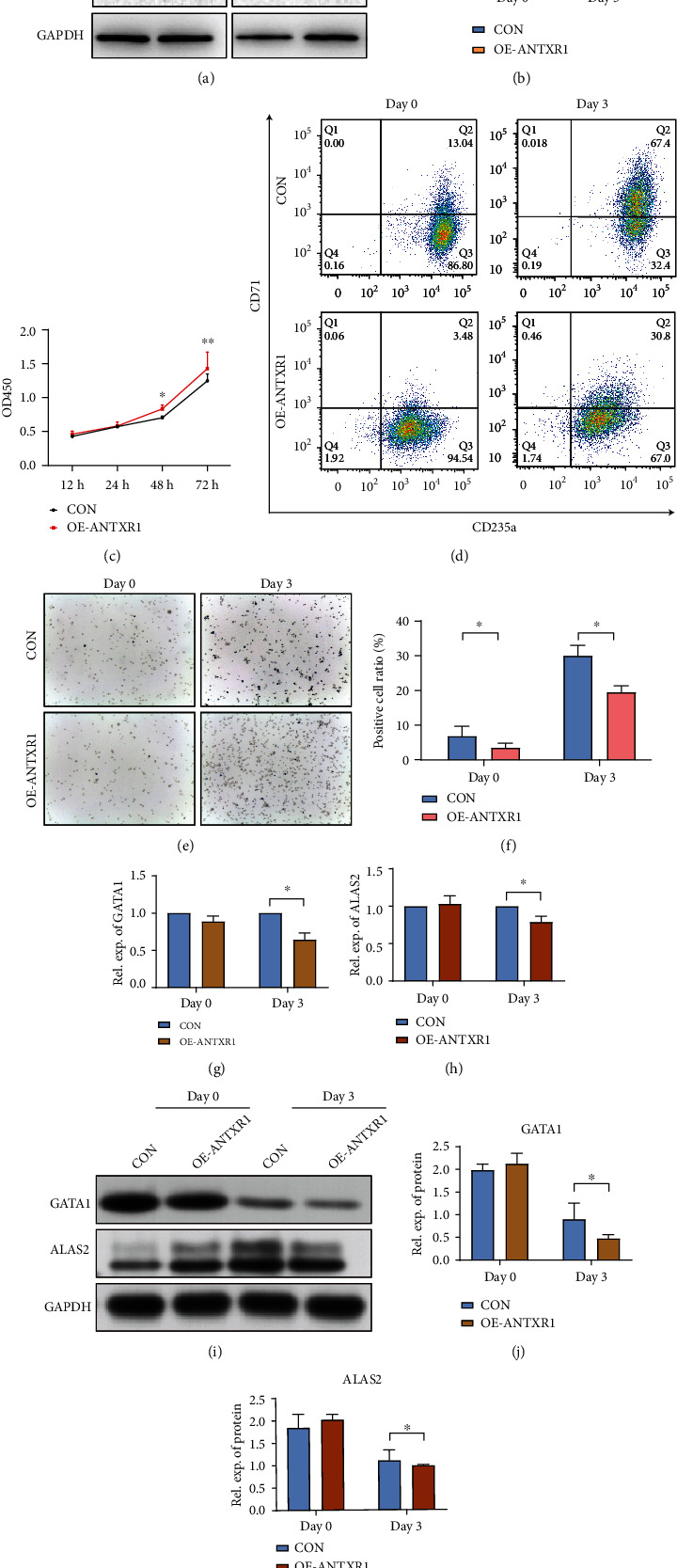
ANTXR1 promotes erythroid cell proliferation and inhibits erythroid cell differentiation and apoptosis in K562 cells. (a) Western blot analysis was used to detect ANTXR1 (45 kDa) expression after transfection of K562 cells with *ANTXR1* overexpression vectors. (b) Quantification of western blots. (c) CCK8 proliferation assay of K562 cells at 12 h, 24 h, 48 h, and 72 h. (d) Flow cytometry detection of CD71 and CD235a expression in K562 erythroid differentiation. (e) Benzidine staining-positive cells. (f) Changes in the proportion of benzidine-positive cells. (g, h) Quantitative reverse transcription polymerase chain reaction (qRT-PCR) was used to determine the mRNA expression levels of GATA1 and ALAS2 in K562 erythrocytes induced by overexpression of ANTXR1. (i) Western blot analysis was used to detect GATA1 (45 kDa) and ALAS2 (65 kDa) protein expression in K562 erythrocytes induced by overexpression of ANTXR1. (j, k) Quantification of western blots. Error bars represent standard deviation of three independent experiments. ^∗^*P* < 0.05, ^∗∗^*P* < 0.01.

To determine the effect of ANTXR1 overexpressing on erythroid differentiation, ANTXR1 was cloned into the pHAGE-fEF-1a-IRES-ZsGreen-2 vector. In the following experiments, the vector was transfected into K562 and cord blood CD34^+^ cells, while cells transfected with an empty vector were used as controls. Western blot was performed to confirm transfection efficiency 48-72 hours after transfection. According to the results, ANTXR1 expression was significantly increased during the induction of erythroid in K562 cells compared with the control group (Figures 1(a) and 1(b)). There was no statistical difference in cell proliferation compared with the control group before 48 hours. Cell proliferation was significantly increased in the OE-ANTXR1 group compared to the control at 48-72 h (Figure 1(c)). ANTXR1 was found to promote proliferation in the study. Flow cytometry analysis showed that the double-positive ratio of overexpression ANTXR1, CD71, and CD235a was significantly reduced compared with the control group, indicating that ANTXR1 inhibited the erythroid differentiation of K562 cells (Figure 1(d)). In comparison to the control group, the proportion of positive cells detected by benzidine staining decreased (Figures 1(e) and 1(f)). In addition, ANTXR1 overexpression inhibited the GATA1 and ALAS2 expression at mRNA and protein levels among the days of D0 to D3 with erythroid differentiation (Figures 1(g)–1(k)).

### 3.2. ANTXR1 Promotes Erythroid Cell Proliferation and Inhibits Erythroid Cell Differentiation and Apoptosis in Cord Blood CD34^+^ Cells

**Figure 2:**
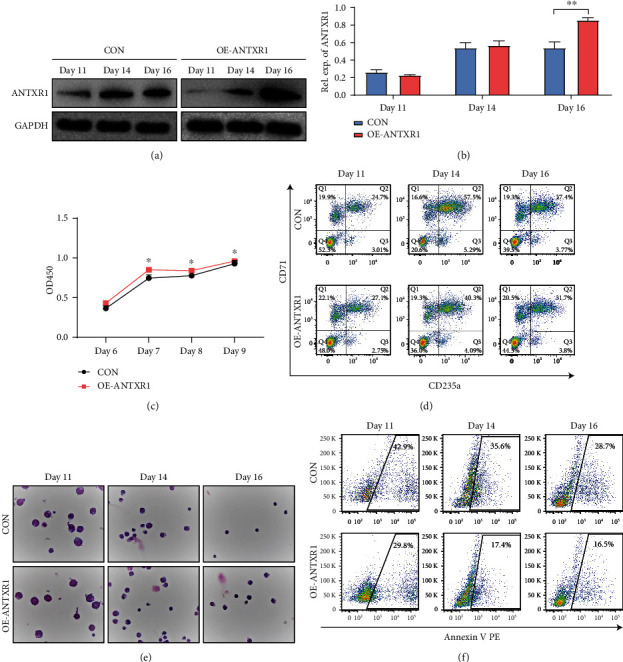
ANTXR1 promotes erythroid cell proliferation and inhibits erythroid cell differentiation and apoptosis in cord blood CD34^+^ cells. (a) Western blot analysis was used to detect ANTXR1 levels after transfection of cord blood CD34^+^ cells with *ANTXR1* overexpression vectors. (b) Quantification of western blots. (c) CCK8 assay of cord blood CD34^+^ cells proliferation at D6, D7, D8, and D9. (d) Flow cytometry detection of CD71 and CD235a expression in cord blood CD34^+^ cells during erythroid differentiation. (e) Wright-Giemsa staining results of cord blood CD34^+^ cells. (f) Flow cytometry to detect the expression of apoptotic cells in the differentiation of cord blood CD34^+^ cells. Error bars represent standard deviation of three independent experiments. ^∗^*P* < 0.05, ^∗∗^*P* < 0.01.

An increase in ANTXR1 expression was observed in cord blood CD34^+^ cells on days 14 and 16 during erythroid induction (Figures 2(a) and 2(b)). CD34 ^+^ cells proliferated from day 7 after ANTXR1 overexpression (Figure 2(c)). According to flow cytometry results, the OE-ANTXR1 showed reduced CD71 and CD235a expression compared with the control group, which indicated that ANTXR1 inhibited the differentiation of cord blood CD34^+^ cells into erythroid (Figure 2(d)). Wright-Giemsa results also showed that ANTXR1 overexpression increased CD34^+^ erythroid cell volume, whereas the cells with nuclear shift and nuclear shrinkage was significantly reduced compared to those in the control group (Figure 2(e)).

Apoptosis experiment results showed a significantly higher apoptosis rate in the CON group (42.9%, 35.6%, and 28.7%) on days 11, 14, and 16 of cord blood CD34^+^ cell differentiation than that in the OE-ANTXR1 group (29.8%, 17.4%, and 16.5%) (Figure 2(f)). The results of these studies confirm that ANTXR1 regulates erythroid proliferation and differentiation in K562 cells and cord blood CD34^+^ cells during erythroid induction.

### 3.3. ANTXR1 Silencing Inhibits Cell Proliferation and Promotes Erythroid Differentiation

**Figure 3:**
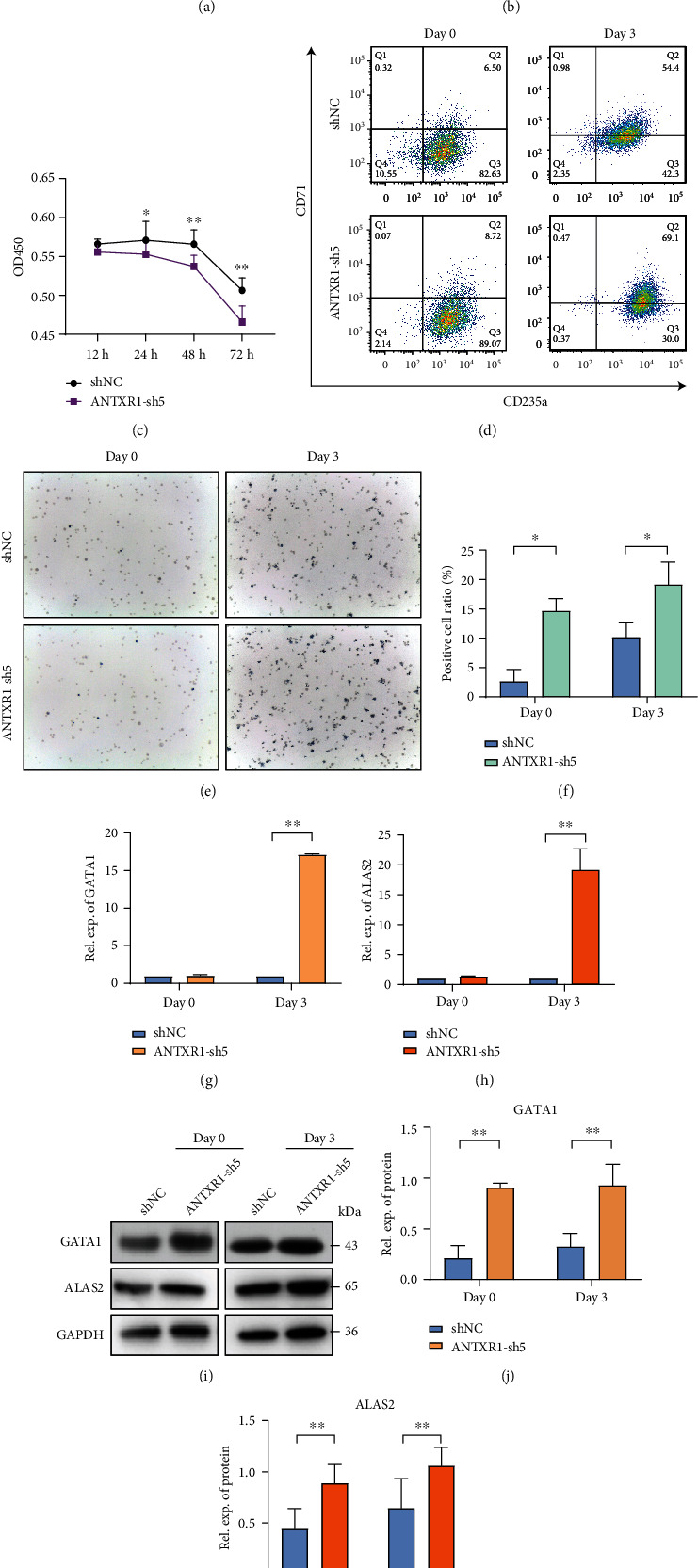
Interfering with ANTXR1 inhibits erythroid cell proliferation and promotes erythroid cell differentiation and apoptosis in K562 cells. (a) Western blot analysis was used to detect ANTXR1 levels after transfection of K562 cells with *ANTXR1* interfering vectors. (b) Quantification of western blots. (c) CCK8 assay of K562 cells proliferation at 12 h, 24 h, 48 h, and 72 h. (d) Flow cytometry detection of CD71 and CD235a expression in K562 cells erythroid differentiation. (e) Benzidine staining-positive cells. (f) Changes in the proportion of benzidine-positive cells. (g, h) Quantitative reverse transcription polymerase chain reaction (qRT-PCR) was used to determine the mRNA expression levels of GATA1 and ALAS2 in K562 erythrocytes induced by interfering with ANTXR1. (i) GATA1 and ALAS2 protein expression in K562 erythrocytes induced by interfering with ANTXR1. (j, k) Quantification of western blots. Error bars represent standard deviation of three independent experiments. ^∗^*P* < 0.05, ^∗∗^*P* < 0.01.

**Figure 4:**
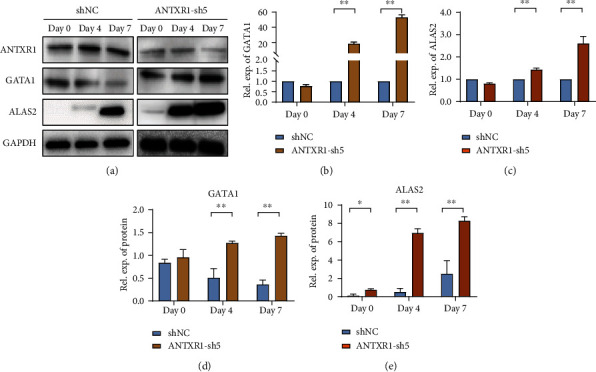
ANTXR1 silencing promotes erythroid cell differentiation in HUDEP-2 cells by interval consecutive analysis. (a) Analysis of GATA1 and ALAS2 protein expressions in HUDEP-2 cells following the knockdown of ANTXR1 the gene expression levels by western blotting and qRT-PCR. (b, c) The relative protein expression levels of GATA1 and ALAS2 were detected after HUDEP-2 cells were transfected with ANTXR1-sh5. (d, e) Quantification of western blots. Error bars represent standard deviation of three independent experiments. ^∗^*P* < 0.05, ^∗∗^*P* < 0.01.

**Figure 5:**
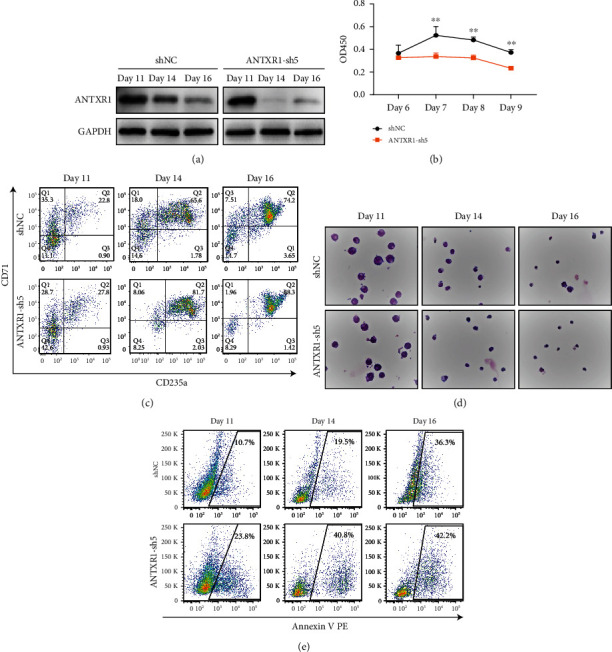
knockdown of ANTXR1 decreased erythroid cell proliferation and promoted erythroid cell differentiation and apoptosis in cord blood CD34^+^ cells. (a) Western blot analysis was used to detect ANTXR1 levels after transfection of cord blood CD34^+^ cells with *ANTXR1* interference vectors. (b) CCK8 assay of cord blood CD34^+^ cells proliferation at D6, D7, D8, and D9. (c) Flow cytometry detection of CD71 and CD235a expression in cord blood CD34^+^ cells during erythroid differentiation. (d) Wright-Giemsa staining results of cord blood CD34^+^ cells. (e) Flow cytometry to detect the expression of apoptotic cells in the differentiation of cord blood CD34^+^ cells. Error bars represent standard deviation of three independent experiments. ^∗^*P* < 0.05, ^∗∗^*P* < 0.01.

To further confirm the role of ANTXR1 in erythroid differentiation, we silenced the ANTXR1 expression in K562, cord blood CD34^+^ cells, and HUDEP-2 cells with short hair RNA (shRNA). After transfection with shRNA, ANTXR1 expression in these three cell lines was significantly reduced compared to the control group cells transfected with shNC (Figures 3(a) and 3(b), 4(a), and 5(a)). ANTXR1 silencing significantly reduced the proliferative capacity of K562 cells after 24 h (Figure 3(c)). The flow cytometry results showed that during erythroid differentiation of K562, the proportion of CD71 and CD235a double-positive cells was significantly increased by interfering with ANTXR1 (Figure 3(d)). The benzidine staining showed that ANTXR1 silencing increased the proportion of the positive cells (Figures 3(e) and 3(f)). In addition, ANTXR1 silencing increased the GATA1 and ALAS2 expressions at mRNA and protein levels from days D0 to D3 throughout the erythroid differentiation (Figures 3(g)–3(k)).

We also found the same GATA1 and ALAS2 expressions trend in HUDEP-2 (Figures 4(a)–4(e)). Similar cell proliferation, differentiation, and apoptosis results were observed in cord blood CD34^+^ cells after ANTXR1 silencing, and CD34^+^ cell proliferation decreased from day 7 (Figure 5(b)). There was a significant increase in CD71 and CD235a expression compared to the shNC group, suggesting that ANTXR1 induced erythroid differentiation in cord blood CD34^+^ cells (Figure 5(c)). The Wright-Giemsa results also showed that during CD34^+^ cells erythroid differentiation, ANTXR1 silencing could induce a smaller volume of cord blood CD34^+^ cells, and the cells with nuclear shift and shrinkage were significantly higher after Day 14 (Figure 5(d)). In addition, the apoptosis rate of shNC group (10.7%, 19.5%, and 36.3%) at days 11, 14, and 16 in cord blood CD34^+^ cells was significantly lower than ANTXR1-sh5 group (23.8%, 40.8%, and 42.2%, respectively (Figure 5(e)). Thus, we speculate that ANTXR1 regulated the proliferation and differentiation of erythroid cells during erythroid induction.

### 3.4. ANTXR1 Regulates Erythroid Cell Differentiation through the Wnt/*β*-Catenin Pathway

**Figure 6:**
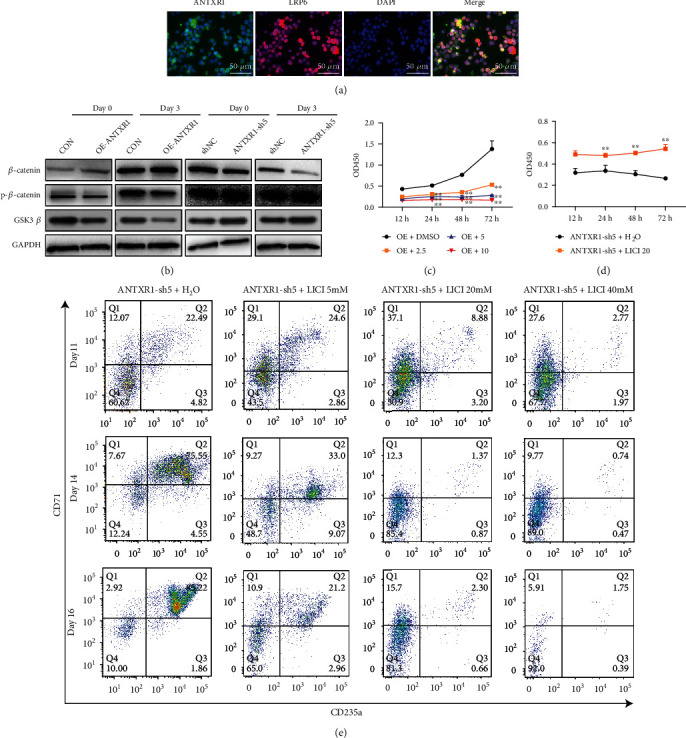
The Wnt/*β*-catenin signaling pathway cooperates with ANTXR1 in regulating erythroid cell differentiation. (a) The expression of ANTXR1 and LRP6 in K562 cells was detected by immunofluorescence with a confocal laser scanning microscope. ANTXR1 was labeled with 428 nm FITC, which represents green; LRP6 was labeled with Texas red; and nuclei were stained by DAPI to compare to the cell membrane. The merged images indicate the localization of ANTXR1 and LRP6 in the cell membrane, which the overlay of green and red give yellow. (b) The protein levels of *β*-catenin (92 kDa), P-*β*-catenin (85 kDa), and GS3*β* (46 kDa) were measured by western blotting after K562 cells were overexpressed or knocked down by ANTXR1. (c) Respectively, with K562 cells overexpressing ANTXR1 treated with 2.5, 5, and 10 *μ*mol/L of XAV939 for 24 h. Cell proliferation was determined by CCK-8. (d) K562 cells knockdown ANTXR1 treated with 20 mM LiCl for 24 h, and cell proliferation was determined by CCK-8. (e) Cord blood CD34^+^ cells knockdown ANTXR1 treated with 5, 20, and 40 mM LiCl for 24 h, respectively. Flow cytometry detection of CD71 and CD235a expression. Error bars represent standard deviation of three independent experiments. ^∗^*P* < 0.05, ^∗∗^*P* < 0.01.

Recent studies have shown that the Wnt signaling pathway induces blood cell maturation by regulating the hematopoietic microenvironment [17]. For instance, overexpression of Axin can block Wnt signaling and further inhibit HSCs proliferation in vitro [15]. Immunofluorescence results showed that ANTXR1 and Wnt upstream protein LRP6 were colocalized on the cell membrane of K562 cells in order to determine whether the Wnt signaling pathway is also involved in the regulation of erythroid differentiation by ANTXR1 (Figure 6(a)). Our previous research shows that ANTXR1 interacted with LRP6 to activate Wnt/*β*-catenin signaling pathway in K562 cell line, but whether this interaction is direct or indirect is still unclear. In this experiment, overexpression of *ANTXR1* upregulated the *β*-catenin expression but down-regulated the P-*β-*catenin and GS3*β* expressions. ANTXR1 knockdown reduced *β*-catenin expression but increased P-*β*-catenin and GS3*β* expressions as demonstrated by western blotting analysis (Figure 6(b)). The Wnt/*β*-catenin pathway may be involved in the ANTXR1 regulation of erythroid differentiation.

To further test and verify the mechanisms of ANTXR1 and Wnt/*β*-catenin in the regulation of erythroid differentiation, different concentrations of XAV939 (a chemical that inhibits the regulation of Wnt pathway transcription factor *β*-catenin) were firstly added to K562 cell line that stably overexpress ANTXR1. Different concentrations of LiCl (a special Wnt activator that functions by inhibiting the activity of GSK3*β*) were also analyzed. Compared to the DMSO control group, cell proliferation significantly decreased with increasing XAV939 concentration in K562 cells (Figure 6(c)). In contrast, the proliferation of cells in the ANTXR1-sh5 + LiCl20 group was significantly increased compared with the control group (Figure 6(d)). We used flow cytometry to investigate further the Wnt/*β*-catenin pathway in the erythroid differentiation of hematopoietic stem cells. After interfering with ANTXR1 in cord blood CD34^+^ cells and adding various LiCl concentrations, the double positivity of CD71 and CD235a decreased with the increasing LiCl dose, showing a dose-effect relationship (Figure 6(e)). Taken together, these observations confirmed that Wnt/*β*-catenin has a role in the regulation of ANTXR1 on erythroid differentiation, proliferation, and differentiation. The specific regulation mechanism needs to be further studied.

## 4. Discussion

The hematopoietic microenvironment is mainly regulated by various cytokines. Hematopoietic cells with disrupted gene expression exhibit a state known as “stressed hematopoiesis,” which causes abnormal hematopoietic cell proliferation due to the increased cellular oxidative stress level and early erythrocyte apoptosis [18–20]. Transplantation of hematopoietic stem cells is the primary treatment for many blood diseases, but it is frequently difficult to obtain sufficient HSCs and transplantable donors for this procedure. In addition, an inefficient hematopoietic reconstruction system is still a fundamental cause of hematopoietic engraftment failure [21, 22]. Our study provides theoretical bases for improving effective erythroid expansion by exploring mechanism that membrane protein ANTXR1 regulates the proliferation and differentiation of erythroid cells through wnt/*β*-catenin signaling pathway for the first time.

ANTXR1 was initially found in tumor vascular endothelial cells and promote tumor vascular endothelial formation [23]. ANTXR1 gene mutation can also cause growth retardation, alopecia, pseudo-anodontia, and GAPO syndrome (GAPOS) [24, 25]. ANTXR1 plays an important role in chondrocyte proliferation, and its overexpression causes chondrocyte apoptosis and matrix mineralization [8]. In addition, ANTXR1 inhibition has been reported to reduce esophageal tumor cell proliferation, block the G0/G1 phase, and promote cell apoptosis [26].

Our findings showed that ANTXR1 overexpression promoted K562 cell proliferation, decreased the erythrocyte transcription factors GATA1 and ALAS2 expressions, and delayed the differentiation and apoptosis of the erythroid cell. On the other hand, ANTXR1 knockdown inhibited the erythroid cells proliferation, promoted the GATA1 and ALAS2 expressions, and inhibited the erythroid cell apoptosis. GATA1 is expressed in the erythroid, and its DNA binding region can recognize the specific gene regulatory region of erythrocytes, which is an essential regulatory factor for erythroid development [27, 28]. ALAS2 is the key enzyme for heme synthesis, which can promote hematopoietic cell differentiation by increasing hemoglobin synthesis [29]. Therefore, we speculated that ANTXR1 was involved in regulating erythroid differentiation. This indicates that ANTXR1 may be a new factor that affects the proliferation and differentiation of erythroid cells.

The Wnt/*β*-catenin pathway plays a role in cell proliferation and cycle changes and the maintenance and differentiation of stem cell self-renewal [30]. A large amount of unphosphorylated catenin will be present in the cytoplasm when the Wnt pathway is activated. Upon entering the nucleus, *β*-catenin binds to transcription factor (TCF) protein and acts as a co-activator of TCF to stimulate transcription of Wnt target genes. Our previous research found that there was an interaction between ANTXR1 and LRP6, the colocalization of ANTXR1 and LRP6 in cell membrane indicated that ANTXR1 regulates erythroid proliferation and differentiation is probably through interacting with the membrane protein LRP6 and activating the Wnt/*β*-catenin signaling pathway [31]. However, it is not clear whether ANTXR1 involved in regulating erythroid proliferation and differentiation by interacting with LRP6. It has been shown that overexpression of Axin to block Wnt signaling can inhibit HSCs proliferation in vitro [16]. The transduction of *β*-catenin into HSCs of transgenic Bcl-2 mice resulted in increased HSCs phenotype during in vitro culture [32]. Overexpression of Wnt inhibitory factors in bone marrow stromal cells or/and osteoblasts can break the quiescent state of HSCs, resulting in impaired HSCs self-renewal and reduced hematopoietic reconstitution in vivo [15, 33, 34]. Activating *β*-catenin signaling with GSK3*β* inhibitors can improve the HSCs expansion in vitro and promote the hematopoietic reconstitution ability of HSCs in vivo [35–37].

Our study shows that ANTXR1 activates the Wnt/*β*-catenin signaling pathway and promotes the proliferation of erythroid cells through inhibit GATA1 and ALAS2 transcription. In contrast, ANTXR1 knockdown reduced the *β*-catenin expression, and Wnt/*β*-catenin signaling was also inhibited, GATA1 and ALAS2 expressions were increased and promote erythroid differentiation. It has therefore been proposed that ANTXR1 may affect the proliferation and differentiation of erythroid cells through the Wnt/*β*-catenin signaling pathway.

Recent studies have found that the Wnt signaling pathway strengthens the differential effect on HSCs self-renewal [38]. The Wnt/*β*-catenin signaling pathway may affect the proliferation and differentiation of hematopoietic stem cells in a dose-response-dependent manner at different levels. Two times the normal level of Wnt pathway activity promotes HSCs proliferation, and more than four times the Wnt pathway activity impairs HSCs self-renewal and proliferation. In this study, overexpression of ANTXR1 increased the activity of Wnt pathway and increased the proliferation of HSCs cells. We speculate that the activity of Wnt pathway may be at a normal low level. Further studies are needed to determine the effect of Wnt pathway activation induced by different expression levels of ANTXR1 on erythrocyte proliferation and differentiation. In addition, STRING database shows that the downstream transcription factor JUN of the Wnt/*β*-catenin pathway interacts with GATA1. As an important protein of the Wnt pathway, Wnt1 and JUN may also interact (https://cn.string-db.org/cgi/network?taskId=bjU1mtYXnzEc&sessionId=bw0iWzp61fNp). Our previous research showed that ANTXR1 regulates *γ*-globin expression is mainly activating the Wnt/*β*-catenin signaling pathway, which initiates the expression of the downstream transcription factor c-Jun. Erythrocyte differentiation and development are accompanied by globin production. Therefore, this study suggests that GATA1/ALAS2, an important transcription factor for hematopoiesis and erythrocyte differentiation, may also interact with the c-Jun gene in Wnt/*β*-catenin to regulate erythrocyte proliferation and differentiation [39, 40]. However, further investigation is needed to determine whether ANTXR1 interacts with Wnt1. The use of gene expression profiles in peripheral blood for the diagnosis of autoimmune diseases, leukemia, and other genetic disorders is becoming increasingly common in clinical settings. The participation of all hematopoietic cells in the hematopoietic system, whether directly or indirectly, is highly dependent on the stage of hematopoietic development.

**Figure 7:**
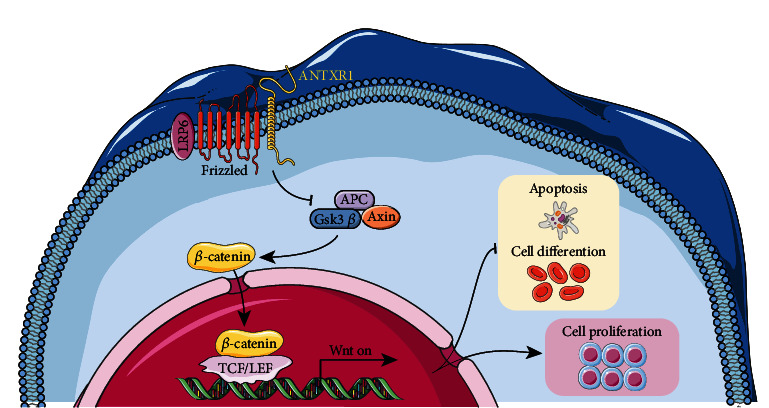
The hypothetical molecular mechanism of ANTXR1 affecting erythroid cell proliferation and differentiation through wnt/*β*-catenin signaling pathway.

In summary, ANTXR1 may play essential roles in regulating the HSCs microenvironment through Wnt signaling pathway (Figure 7) that may indirectly trigger Axin1, DKK1, and Wif1 [41–43]. It requires further understanding of ANTXR1 in regulating hematopoiesis in a gene network. In conclusion, due to the complex mechanism of Wnt pathway regulation of HSCs self-renewal and maintenance, ANTXR1 modulates erythroid cell proliferation and differentiation, but many unknowns are still to be revealed. The findings of this research might provide a novel approach for treating blood disorders and improve the proliferation of hematopoietic stem cells.

## Figures and Tables

**Table 1:** List of primers used for RT-qPCR assays.

Gene	Primer sequence (5′-3′)
GAPDH	F: GCACCGTCAAGGCTGAGAAC
	R: TGGTGAAGACGCCAGTGGA
ANTXR1	F: CGGATTGCGGACAGTAAGGAT
	R:TCCTCTCACGACAACTTGAAATG
GATA1	F: CTGTCCCCAATAGTGCTTATGG
	R:GAATAGGCTGCTGAATTGAGGG
ALAS2	F: CCAAACAGGAACTGGTGAGTC
	R:TCATTCGTTCGTCCTCAGTG

## Data Availability

The data used to support the findings of this study are included within the article.

## Abbreviations

EF: Edema factor
LF: Lethal factor
ANTXR1: Anthrax toxin receptor 1
HSCs: Hematopoietic stem cells
MEPs: Megakaryocytic erythroid progenitors
CMPs: Common myeloid progenitor cells
BFU-E: Erythroid blast-forming units
CFU-E: Erythroid colony-forming units
KLF1: Krüppel-like factor 1
SNP: Single-nucleotide polymorphism
CB: Cord blood
HbF: Fetal hemoglobin
FBS: Fetal bovine serum
HUDEP-2: Human umbilical cord blood-derived erythroid progenitor-2
GATA1: GATA-binding factor 1 or GATA-1 (also termed erythroid transcription factor)
ALAS2: 5'-Aminolevulinate synthase 2
GAPOS: GAPO syndrome
TCF: Transcription factor
shRNA: Short hair RNA.

## References

[1] Hawley R. G., Ramezani A., Hawley T. S. (2006). Hematopoietic stem cells. *Methods Enzymol*.

[2] Wakefield E. O., Pantaleao A., Popp J. M. (2018). Describing perceived racial bias among youth with sickle cell disease. *Journal of Pediatric Psychology*.

[3] Hussein N., Weng S., Kai J., Qureshi N. (2015). Preconception risk assessment for thalassaemia, sickle cell disease, cystic fibrosis and Tay-Sachs disease. *Cochrane Database Systematic Reveviews*.

[4] Fernández J. J., García D. R. C., Fernández-Urién I. (2017). Mediterranean lymphoma, an uncommon case of iron⁃deficiency anemia. *Acta gastro-enterologica Belgica*.

[5] Gao M. Y., Zheng Y. Z. (2020). Research progress on transcriptional regulation mechanism of erythropoiesis. *International Journal of blood transfusion and hematology*.

[6] Nanda A., Carsonwalter E. B., Seaman S. (2004). TEM8 interacts with the cleaved C5 domain of collagen alpha 3(VI). *Cancer Research*.

[7] Werner E. A., Kowalczyk P., Faundez V. (2006). Anthrax toxin receptor 1/tumor endothelium marker 8 mediates cell spreading by coupling extracellular ligands to the actin cytoskeleton. *Journal of Biological Chemistry*.

[8] Jiang Q., Qin X., Yoshida C. A. (2020). Antxr1, which is a target of Runx2, regulates chondrocyte proliferation and apoptosis. *International Journal of Molecular Sciences*.

[9] Chaudhary A., Hilton M. B., Seaman S. (2012). TEM8/ANTXR1 blockade inhibits pathological angiogenesis and potentiates tumoricidal responses against multiple cancer types. *Cancer Cell*.

[10] Vathipadiekal V., Farrell J. J., Wang S. (2016). A candidate transacting modulator of fetal hemoglobin gene expression in the Arab-Indian haplotype of sickle cell anemia. *American Journal of Hematology*.

[11] Al-Ali Z. A., Fallatah R. K., Aljaffer E. A. (2018). ANTXR1 intronic variants are associated with fetal hemoglobin in the Arab Indian haplotype of sickle cell disease. *Acta Haematol*.

[12] Xu Z., Robitaille A. M., Berndt J. D. (2016). Wnt/*β*-catenin signaling promotes self-renewal and inhibits the primed state transition in naïve human embryonic stem cells. *Proceedings of the National Academy of Sciences*.

[13] Zhao C., Blum J., Chen A. (2007). Loss of beta-catenin impairs the renewal of normal and CML stem cells in vivo. *Cancer Cell*.

[14] Fleming H. E., Janzen V., Celso C. L. (2008). Wnt signaling in the niche enforces hematopoietic stem cell quiescence and is necessary to preserve self-renewal in vivo. *Cell Stem Cell*.

[15] Schaniel C., Sirabella D., Qiu J., Niu X., Lemischka I. R., Moore K. A. (2011). Wnt-inhibitory factor 1 dysregulation of the bone marrow niche exhausts hematopoietic stem cells. *Blood*.

[16] Reya T., Duncan A. W., Ailles L. (2003). A role for Wnt signalling in self-renewal of haematopoietic stem cells. *Nature*.

[17] Hotra S., Kincade P. W. (2009). Wnt-related molecules and signal pathway equilibrium in hematopoiesis. *Cell Stem Cell*.

[18] Tian X., Cong F., Guo H., Fan J., Chao G., Song T. (2019). Downregulation of Bach1 protects osteoblasts against hydrogen peroxide-induced oxidative damage in vitro by enhancing the activation of Nrf2/ARE signaling. *Chemico-Biological Interactions*.

[19] Cao Y., Fang Y., Cai J. (2016). ROS functions as an upstream trigger for autophagy to drive hematopoietic stem cell differentiation. *Hematology*.

[20] Taher A. T., Weatherall D. J., Cappellini M. D. (2018). Thalassaemia. *Lancet*.

[21] Seggewiss R., Einsele H. (2010). Immune reconstitution after allogeneic transplantation and expanding options for immunomodulation: an update. *Blood*.

[22] Pineault N., Boyer L. (2011). Cellular-based therapies to prevent or reduce thrombocytopenia. *Transfusion*.

[23] Besschetnova T. Y., Ichimura T., Katebi N., Croix B. S., Bonventre J. V., Olsen B. R. (2015). Regulatory mechanisms of anthrax toxin receptor 1-dependent vascular and connective tissue homeostasis. *Matrix Biology*.

[24] Bayram Y., Pehlivan D., Karaca E. (2014). Whole exome sequencing identifies three novel mutations in ANTXR1 in families with GAPO syndrome.

[25] Chen D., Bhat-Nakshatri P., Goswami C., Badve S., Nakshatri H. (2013). ANTXR1, a stem cell-enriched functional biomarker, connects collagen signaling to cancer stem-like cells and metastasis in breast cancer. *Cancer Research*.

[26] Ren Y., He W., Qi M. L., Chen X. W., Liang L. W., Feng J. M. (2016). Effect of silencing antxr1 gene by RNA interference on proliferation and apoptosis of esophageal cancer Eca109 cells. *South China Journal of National Defense Medicine*.

[27] Luo C. Y., Wang J. W. (2008). GATA-1 and erythropoiesis. *International Journal of Internal Medicine*.

[28] Gutiérrez L., Nikolic T., van Dijk T. B. (2007). Gata l regulates dendritic-cell development and survival. *Blood*.

[29] Rio S., Gastou M., Karboul N. (2019). Regulation of globin-heme balance in diamond-Blackfan anemia by HSP70/GATA1. *Blood*.

[30] Miller J. R. (2002). The Wnts. *Genome Biology*.

[31] Haack F., Köster T., Uhrmacher A. M. (2021). Receptor/raft ratio is a determinant for LRP6 phosphorylation and WNT/*β*-catenin signaling. *Frontiers in Cell and Developmental Biology*.

[32] Ming M., Wang S., Wu W. (2012). Activation of Wnt/*β*-catenin protein signaling induces mitochondria-mediated apoptosis in hematopoietic progenitor cells. *Journal of biological chemistry*.

[33] Nemeth M. J., Bodine D. M. (2007). Regulation of hematopoiesis and the hematopoietic stem cell niche by Wnt signaling pathways. *Cell Research*.

[34] Gattinoni L., Zhong X. S., Palmer D. C. (2009). Wnt signaling arrests effector T cell differentiation and generates CD8^+^ memory stem cells. *Nature Medicine*.

[35] Trowbridge J. J., Xenocostas A., Moon R. T., Bhatia M. (2006). Glycogen synthase kinase-3 is an in vivo regulator of hematopoietic stem cell repopulation. *Nature Medcine*.

[36] Ko K. H., Holmes T., Palladinetti P. (2011). GSK-3beta inhibition promotes engraftment of ex vivo-expanded hematopoietic stem cells and modulates gene expression. *Stem Cells*.

[37] Huang J., Nguyen-McCarty M., Hexner E. O., Danet-Desnoyers G., Klein P. S. (2012). Maintenance of hematopoietic stem cells through regulation of Wnt and mTOR pathways. *Nature Medicine*.

[38] Luis T. C., Naber B. A., Roozen P. P. (2011). Canonical wnt signaling regulates hematopoiesis in a dosage-dependent fashion. *Cell Stem Cell*.

[39] Dan R., O'Brien T. G. (1998). AP-1 activity affects the levels of induced erythroid and megakaryocytic differentiation of K562 cells. Archives of Biochemistry & Biophysics. *Archives of Biochemistry & Biophysics*.

[40] Elagib K. E., Xiao M., Hussaini I. M. (2004). Jun blockade of erythropoiesis: role for repression of GATA-1 by HERP2. *Molecular & Cellular Biology*.

[41] Goessling W., North T. E., Loewer S. (2009). Genetic interaction of PGE2 and Wnt signaling regulates developmental specification of stem cells and regeneration. *Cell*.

[42] Nostro M. C., Cheng X., Keller G. M., Gadue P. (2008). Wnt, activin, and BMP signaling regulate distinct stages in the developmental pathway from embryonic stem cells to Blood. *Cell Stem Cell*.

[43] Singbrant S., Karlsson G., Ehinger M. (2010). Canonical BMP signaling is dispensable for hematopoietic stem cell function in both adult and fetal liver hematopoiesis, but essential to preserve colon architecture. *Blood, The Journal of the American Society of Hematology*.

